# Mobile diagnostics and consultation for the prevention of the metabolic syndrome and its secondary diseases in Brandenburg—study protocol of a regional prospective cohort study: the Mobile Brandenburg Cohort

**DOI:** 10.1186/s40814-021-00898-w

**Published:** 2021-08-30

**Authors:** Anne Schraplau, Andrea Block, Andreas Häusler, Pia-Maria Wippert, Michael A. Rapp, Heinz Völler, Klaus Bonaventura, Frank Mayer

**Affiliations:** 1 University Outpatient Clinic, Sports Medicine and Sports Orthopedics, University of Potsdam, Am Neuen Palais 10, 14469 Potsdam, Germany; 2 Faculty of Health Sciences Brandenburg, University of Potsdam, Potsdam, Germany; 3 Medical Sociology and Psychobiology, University of Potsdam, Potsdam, Germany; 4 Social and Preventive Medicine, University of Potsdam, Potsdam, Germany; 5 Rehabilitation Medicine, University of Potsdam, Potsdam, Germany; 6 Klinik am See, Rehabilitation Center for Internal Medicine, Rüdersdorf, Germany; 7 Internal Medicine/Cardiology, Ernst-von-Bergmann Clinic, Potsdam, Germany

**Keywords:** Metabolic syndrome, Mobile diagnostics, Prevention, Nutrition, Physical activity, Rural health

## Abstract

**Background:**

The metabolic syndrome (MetS) is a risk cluster for a number of secondary diseases. The implementation of prevention programs requires early detection of individuals at risk. However, access to health care providers is limited in structurally weak regions. Brandenburg, a rural federal state in Germany, has an especially high MetS prevalence and disease burden. This study aims to validate and test the feasibility of a setup for mobile diagnostics of MetS and its secondary diseases, to evaluate the MetS prevalence and its association with moderating factors in Brandenburg and to identify new ways of early prevention, while establishing a “Mobile Brandenburg Cohort” to reveal new causes and risk factors for MetS.

**Methods:**

In a pilot study, setups for mobile diagnostics of MetS and secondary diseases will be developed and validated. A van will be equipped as an examination room using point-of-care blood analyzers and by mobilizing standard methods. In study part A, these mobile diagnostic units will be placed at different locations in Brandenburg to locally recruit 5000 participants aged 40-70 years. They will be examined for MetS and advice on nutrition and physical activity will be provided. Questionnaires will be used to evaluate sociodemographics, stress perception, and physical activity. In study part B, participants with MetS, but without known secondary diseases, will receive a detailed mobile medical examination, including MetS diagnostics, medical history, clinical examinations, and instrumental diagnostics for internal, cardiovascular, musculoskeletal, and cognitive disorders. Participants will receive advice on nutrition and an exercise program will be demonstrated on site. People unable to participate in these mobile examinations will be interviewed by telephone. If necessary, participants will be referred to general practitioners for further diagnosis.

**Discussion:**

The mobile diagnostics approach enables early detection of individuals at risk, and their targeted referral to local health care providers. Evaluation of the MetS prevalence, its relation to risk-increasing factors, and the “Mobile Brandenburg Cohort” create a unique database for further longitudinal studies on the implementation of home-based prevention programs to reduce mortality, especially in rural regions.

**Trial registration:**

German Clinical Trials Register, DRKS00022764; registered 07 October 2020—retrospectively registered.

## Background

The burden of non-communicable diseases is increasing worldwide and is among the leading causes of death [1]. The metabolic syndrome (MetS) is one of the main risk factor clusters for the incidence of metabolic and cardiovascular diseases (e.g., type 2 diabetes mellitus [2], non-alcoholic fatty liver disease [3], myocardial infarction and stroke [4]), and is also associated with the development of musculoskeletal disorders (e.g., osteoarthritis [5], tendinopathy [6]) and neuropsychiatric dysfunctions (e.g., dementia [7], depression [8]). MetS is characterized by dyslipidemia, high blood pressure, elevated blood glucose levels, and central obesity [9], and it is strongly linked to an increase in morbidity and all-cause mortality [4]. The worldwide prevalence of MetS is estimated to be around 25% [10] with an overall prevalence of up to 33% in some countries, e.g., the USA [11]. Therefore, the increasing prevalence of MetS and its secondary diseases is of considerable medical and economic relevance.

Rural regions carry a higher disease burden and have poorer health outcomes than the urban population [12]. In the USA, rural areas demonstrate a higher type 2 diabetes mellitus prevalence [13] and an elevated probability of hospital mortality compared to urban regions [14]. In comparison with other German federal states, Brandenburg, a rural and structurally weak region, demonstrates a higher type 2 diabetes mellitus prevalence with rates of up to 14.5% [15, 16]. According to the German Heart Report 2018, the mortality rate due to acute myocardial infarction in the state of Brandenburg is the highest in Germany [17]. Since the in-patient treatment of myocardial infarction in Brandenburg does not differ from that in urban regions [18], the increased mortality could be due to a high burden of risk factors and deficits in the early detection of individuals at risk. With an overall MetS-prevalence of 22% published by Moebus et al. in 2008, Brandenburg had the third highest age-standardized prevalence rate of this important risk factor complex nationwide [19]. The variations between urban and rural areas may be explained by demographic and socio-economic differences or environmental influences. Further, restricted accessibility of health care providers due to limited infrastructure, increased individual traveling times, as well as the acute shortage of physicians in rural areas may contribute to the variation [12, 13, 20–22]. It is therefore necessary to evaluate the specific causes and develop innovative strategies to overcome these challenging structural circumstances [21, 23].

Different approaches and distance-bridging procedures are currently discussed or tested. For example, by offering and coordinating individual (autonomous) traffic or structured shared transportation to the health care facilities, patient mobility is improved [24]. Furthermore, home visits realized by medical assistants and health care professionals was evaluated as a good opportunity to delegate medical care and supporting general practitioners to provide more capacity within practices and outpatient clinics [25]. Moreover, web-based consultations and telemedicine approaches, possibly in combination with home visits, could be suitable for treatment and medical care [24]. In addition, mobile physicians’ offices were rated as an appropriate alternative to local family practices and to reach patients and offer medical care in rural areas [20].

However, all these options require an individual to have been previously identified as at risk and in need of medical consultation. Therefore, only patients who have already been admitted to the health care system either because of existing diagnosis or at least conspicuous symptoms and complaints, or those who have an intrinsic motivation and awareness for medical prevention are reached. However, an earlier identification of individuals at risk is needed to effectively prevent the onset and progression of diseases.

Therefore, a decentralized time-saving and cost-effective early detection of risk factor profiles such as the MetS and its secondary diseases is deemed necessary. A mobile diagnostic procedure may be an effective approach to ensure a local, near-to-home initial screening for risk profiles at an early stage (also) in parts of the population that are insufficiently covered by existing health care structures. Patients can then be referred to appropriate health care providers and prevention, treatment, or therapy programs. Lifestyle modifications including dietary and physical activity interventions are especially efficient and cost-effective in primary and secondary prevention as well as treatment of MetS and its pathophysiological consequences [26–28]. Thus, a mobile prevention strategy, combining mobile diagnostics and the implementation of home-based lifestyle interventions, could be a promising approach.

Therefore, the aims of this study are to (1) develop and assess the reliability and validity of a setup for mobile screening and diagnostic of MetS and its secondary diseases in a pilot study, (2) test the concept and feasibility of this approach for mobile and close-to-home early diagnostic of MetS (main study part A) and its secondary diseases (main study part B) in a rural area of Germany (federal state of Brandenburg), (3) determine the prevalence of MetS (part A) and previously undiagnosed secondary diseases (part B), (4) examine any relationships of MetS with (possibly not yet established) causal, moderating and risk factors (socio-economic, lifestyle, clinical, physiological, genetic parameters) in the large (rural) sample, and (5) thereby develop a “Mobile Brandenburg Cohort” to allow future longitudinal studies. The mobile diagnostic setup and the developed “Mobile Brandenburg Cohort” will be the basis for subsequent follow-up approaches and the implementation of suitable home-based prevention strategies to reduce the incidence and prevalence of MetS and its secondary diseases.

## Methods/design

### Study design and flow

**Fig. 1:**
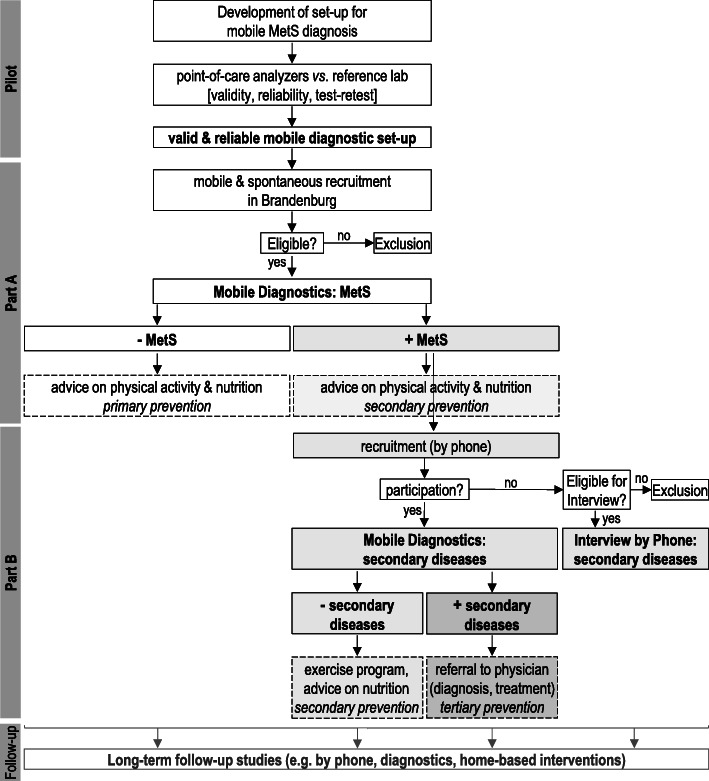
Study design and flow. In the pilot study, the mobile diagnostic set-up is developed; point-of-care-analyzers are tested for validity and reliability versus a reference lab. The mobile set-up for a valid and reliable diagnosis of the metabolic syndrome (MetS) is applied in the study part A. The mobile examination unit is placed mainly at rural places across Brandenburg, and participants are recruited on-site. After inclusion, participants are examined for MetS and receive advices on physical activity and healthy nutrition for prevention of MetS and its secondary diseases. A subgroup of participants with MetS diagnosed in part A is recruited for a mobile examination regarding secondary diseases. Participants receive advices on nutrition and an exercise program at hand and they are referred to physicians for further diagnosis and treatment, if needed. Persons who are not able to participate to the mobile examination of part B are interviewed via telephone. A “Mobile Brandenburg Cohort” is established and can be continued in long-term follow-up studies

After development and validation of mobile laboratory and diagnostic units for a van and a truck (pilot study), a prospective cohort study is to be implemented: main study “part A” is a cross-sectional study, in which baseline measurements will be performed, and MetS prevalence and associated factors are to be evaluated by screening participants in a mobile diagnostic unit near to the participant’s homes. In “part B,” persons previously diagnosed with MetS in part A will be examined (mobile medical examination in a truck or phone-based interviews) to assess secondary diseases of MetS (Fig. 1). The feasibility of the mobile diagnostic approach (part A and B) is to be evaluated. All parts of the study will form the basis for the establishment of the “Mobile Brandenburg Cohort,” which will allow further longitudinal studies.

In the pilot study, setups for the mobile diagnosis of MetS (for part A) and its secondary diseases (for part B) are to be developed. On that purpose, standard methods will be transferred from clinical, stationary routines into a van or small truck that will be thus equipped like a medical examination room to set up a mobile diagnostic unit. Mobile MetS diagnosis using point-of-care analyzers will be tested for validity and reliability.

In part A, the mobile examination unit is placed at different sites in Brandenburg. Participants will be recruited locally and examined for MetS in a physician-independent, mobile setting by a health care professional. Socio-economic status, stress perception, life events, and physical activity will be assessed by approved questionnaires. The participants will receive a short, flyer-supported consultation on healthy nutrition and recommended physical activity to either prevent MetS (primary prevention) or to prevent the development of diseases (secondary prevention). The whole MetS screening and assessing of all parameters is to be limited to approximately 15 min.

In part B, participants diagnosed with MetS in part A will be recruited for a later detailed examination to check for indications of secondary diseases of MetS. Part B examinations will be also done in a mobile examination unit close to the participant’s home by a health care professional together with a physician. A detailed medical history, a clinical examination and instrumental as well as questionnaire-supported diagnostics for internal, orthopedic, cardiological, and neurological-psychiatric abnormalities will be carried out. In addition, the participants will receive a detailed consultation on healthy nutrition and a prevention exercise program directly at hand. The whole mobile clinical examination and consultation of part B will take no more than approximately 60 min. Participants who will be not eligible to mobile examination for secondary diseases will be invited to a telephone interview comprising an assessment with questionnaires that provides comparable and advanced information to the part B examinations, if procurable.

In all study parts, the participants will get their test results instantly and, if necessary due to MetS diagnosis and abnormal parameters, they will be referred to their general practitioner or a responsible medical specialist for further diagnostics and treatment.

By collecting data on MetS and its secondary diseases using mobile diagnostic units at various locations throughout the state of Brandenburg, a “Mobile Brandenburg Cohort” is to be established which can be used for further follow-up measurements with a longitudinal approach.

### Participants

To be eligible for part A, adults (men and women) with an age between 40 and 70 years will be recruited. As the study aims to implement prevention strategies to reduce morbidity and mortality of the population due to MetS, the target population for inclusion are persons with emerging risk for MetS, but less probable presence of (multiple) manifested secondary diseases. Therefore, the age range was chosen to reach participants within the working and retirement age that feature a number of age-related risks for the presence of MetS and for development of its secondary diseases [29, 30]. To be eligible for part B, participants have to be diagnosed with MetS in part A, but have to have no (known and diagnosed) secondary disease. To be eligible for all study parts, participants have to be able to understand the study information and must provide written informed consent for each part.

### Recruitment strategy

In part A, a passive recruitment strategy will be applied with a target population of all residents of Brandenburg that voluntarily approaches to the mobile diagnostic units [31]. Participants will be recruited at different locations in Brandenburg by setting up the mobile diagnostic unit in mainly rural areas far from metropolitan regions. In order to reach as many people as possible, targeted public relations work will be carried out and the mobile study unit is to be placed at market places and at events such as health days and festivals. Cooperation with companies and public administrations is planned in order to make the study known to their employees and encourage them to participate. Announcements will be done by advertising posters, articles in local newspapers and internal newsletters in companies, reports at local radio stations, and at online event calendars of communities and towns. On site, interested passers-by and participants will be directly addressed and recruited spontaneously.

For part B, potentially eligible participants who were diagnosed with MetS in part A and give consent to be re-contacted, will be recruited by telephone. Eligible persons will be allocated to the mobile examination or telephone interview in study part B.

### Sample size determination

For the pilot study (test-retest, reliability), 50 participants will be recruited. For part A and part B, sample size determination was based on the results of Moebus et al. (2008), a nationwide study on primary care patients that revealed an age-standardized MetS prevalence of 22% in Brandenburg [19]. The aim is to recruit 5000 participants in part A to develop the “Mobile Brandenburg Cohort,” and to identify approximately 1100 participants with MetS who are potentially eligible for later recruitment for part B.

### Diagnosis of metabolic risk factors

In all study parts, the MetS diagnosis is based on the definition by Alberti et al. [9] (Table 1). The mobile diagnostic approach requires spontaneous recruitment throughout the day and participants will not be always in a fasted state prior to blood sampling. Therefore, the cut-off point for elevated blood glucose is adapted according to Schipf et al. [32]. For the lipid profile, it was shown that there is little difference between fasting and non-fasting values [33] and for the estimation of an initial risk and for MetS diagnosis, also non-fasting lipids are acceptable [34]. Moreover, the more stable and meal-independent glycated hemoglobin (Hb1Ac) will be determined. It provides additional hints about impaired long-term regulation of glucose metabolism as well as helps to meaningfully assess metabolic risk factors and lipid profile even in a non-fasted state [34].

**Table 1:** Criteria for diagnosis of the metabolic syndrome according to Alberti et al. [9]

The presence of any 3 of the following 5 risk factors constitutes a diagnosis of MetS	
Measure	Cut points/criteria
Elevated blood pressure	Systolic ≥ 130 mmHg and/or diastolic ≥ 85 mmHg [or antihypertensive drug treatment]
Elevated triglycerides	≥ 150 mg/dl [or drug treatment for elevated triglycerides]
Reduced HDL-cholesterol	Males: < 40 mg/dl, females: < 50 mg/dl [or drug treatment for reduced HDL-cholesterol]
Elevated blood glucose	≥ 100 mg/dl (fasting) or ≥ 144 mg/dl (non-fasting)* [or drug treatment for elevated glucose/type 2 diabetes mellitus]
Elevated waist circumference	Population-/country-specific, according to IDF recommendation for people with European origin: males ≥ 94 cm, females ≥ 80 cm

Abbreviations: *IDF* International Diabetes Federation, *HDL* high density lipoprotein, *MetS* metabolic syndrome

*Adaption to non-fasting blood samples according to Schipf et al. [32]

### Procedures

An overview of the methods used and parameters measured in part A and part B is presented in Table 2.

**Table 2:** Overview of conducted measurements during the mobile examinations and the telephone interview

**Part A**	
- Metabolic syndrome diagnosis (ref. Table 1) - Glycated hemoglobin (Hb1Ac) - Pre-existing diseases and drug treatment - Body weight and body height - Socio-demographic basic information - Perceived stress (PSS-4^a^) - Life events within the last 3 months [35] - Physical activity (IPAQ-Short Form^b^)	
**Part B [mobile examination]**	**Part B [interview by phone]**
- Metabolic syndrome diagnosis (ref. Table 1)	
- Glycated hemoglobin (Hb1Ac)	
- Venous blood sample (for further analysis)	
- Body weight and body height	
- Skinfold thickness (body composition)	
- Clinical examination	
- Resting and exercise electrocardiogram	
- Pre-existing diseases and drug treatment	- Comorbidity (CCI^e^)
- Medical history	- Medical history
- Smoking (self-reported; frequency, quantity)	- Smoking (self-reported; frequency, quantity)
- Alcohol consumption (self-reported; frequency, quantity [36])	- Alcohol consumption (self-reported; frequency, quantity [36])
- Depression (PHQ-9^c^)	- Depression (modified PHQ-8^f^)
- Mild cognitive impairment (MoCA^d^)	- Amnestic mild cognitive impairment (TICS-M^g^)
	- Urbanity [37]
	- Social support (BSSS^h^)
	- Perceived stress (PSS-10^i^)
	- Childhood trauma (CTS^j^)
	- Affect (PANAS^k^)
	- Psychological well-being (PWB^l^)
	- Musculoskeletal complaints (CPG^m^, BPI^n^)
	- Risk of developing chronic pain (RSI^o^)
	- Utilization of health system [38, 39]
	- Health behavior after part A

^a^Perceived Stress Scale 4 [40]

^b^International Physical Activity Questionnaire [41]

^c^Patient Health Questionnaire 9 [42]

^d^Montreal Cognitive Assessment [43]

^e^Charlson Comorbidity Index [44]

^f^Patient Health Questionnaire 8 [45]

^g^Modified Telephone Interview for Cognitive Status [46]

^h^Berlin Social Support Scales [47]

^i^Perceived Stress Scale 10 [40]

^j^Childhood Trauma Screener [48]

^k^Positive and Negative Affect Schedule [49]

^l^Psychological Well-being Scale [50]

^m^Chronic Pain Grade Scale [51]

^n^Brief Pain Inventory [52, 53]

^o^Risk Stratification Index [35]

### Medical history

In part A, pre-existing conditions and diseases are recorded by asking about current medication to treat type 2 diabetes mellitus, hypertriglyceridemia, hypercholesterolemia, and/or hypertension, which are also inherent part of the MetS diagnosis. In addition, it is asked whether an acute or chronic coronary syndrome, stroke, coronary heart disease, renal insufficiency, or arthritis have already been diagnosed.

In part B, a comprehensive medical history (i.e., previous internal, orthopedic, cardiological, vegetative abnormalities and diseases, operations, medications, family history) as well as clinical examinations (internal medicine, orthopedic) is carried out by the physician.

### Blood sampling and analysis

In all study parts, a blood sample (approx. 70 μl) is collected with capillary tubes after puncturing the finger bulb using safety lancets. Blood glucose as well as blood lipids (triglycerides, high-density lipoprotein cholesterol (HDL)) is analyzed simultaneously using a portable point-of-care analyzing device. During the pilot study, three different commercially available portable point-of-care analyzers (Alere Cholestech LDX, Alere GmbH, Germany; Samsung LABGEO, Samsung Healthcare, South Korea; CardioChek, PTS Diagnostics, USA) are initially compared and validated versus a certified reference laboratory including analysis of test-retest reliability and variability. The measurement device with the best reliable results for blood parameter analysis and MetS diagnosis is picked for further usage during part A and part B. Hb1Ac is also measured by using a point-of-care analyzer (Quo-Test A1C Test Kit, EKF-diagnostic GmbH, Germany).

In part B, blood samples are additionally taken from an antecubital forearm vein using a disposable needle and vacutainer. Blood is centrifuged immediately and serum samples are stored cooled for later additional analyses in a stationary laboratory, if necessary.

### Blood pressure measurement and anthropometrics

In all study parts, the systolic and diastolic blood pressure is measured using a manual sphygmomanometer and a stethoscope, mainly on the left arm, in the seated position after 5 min rest. Measurement of waist circumference is performed by using a non-stretching measuring tape and by obtaining the midpoint between the lowest rib and the top of the iliac crest of the unclothed upper body according to WHO guidelines [54]. The body weight is assessed using an electronic digital scale.

In part B, the body composition is estimated after measuring skinfold thickness using Harpenden calipers (Baty International, UK). Body fat percentage is calculated using ten sites with the equation from Parizkova et al. [55] and using four sites with the method according to Durnin et al. [56]. All measurements are done by a trained health care professional.

### Resting and exercise electrocardiogram

In part B, a bike ergometer (Ergometer ebike comfort, ergoline GmbH, Germany) with the Custo cardio 200 system (Custo med GmbH, Germany) are placed into the van enabling the conduction of a 12-lead resting and exercise electrocardiogram (ECG). A resting ECG is performed while the participant is sitting on the bike ergometer. The protocol for the exercise ECG includes the following: start cycling at 50 W and increase of the workload with 25 or 50 W every 3 min, termination because of exhaustion or other complaints of the participant. After the exercise ECG, the participant continues cycling for 3 min at 50 W and afterwards stays sitting on the bike for two additional minutes while heart frequency and blood pressure is checked.

### Questionnaires for assessment of socio-economic data, physical activity, and psychometric data

In part A, all participants are asked to fill in self-administered paper-pencil questionnaires about their sociodemographic basic information, perceived stress during the last month (PSS-4) [40], questions about life events within the last 3 months [35], and the physical activity (IPAQ-Short Form) [41]. After detailed instruction by a member of the study team, the IPAQ-Short Form is completed independently by the participant. A member of the study staff is always available to answer any questions that may arise during the completion of the questionnaires.

In part B, a self-administered paper-pencil questionnaire is filled in to screen for presence and severity of depression (PHQ-9) [42]. The “Montreal Cognitive Assessment” (MoCA) [43] is used by the trained physician for detection of mild cognitive impairment. The telephone interview of part B, for participants who are not eligible to the mobile examination, comprises questions about medical history, life style factors (self-reported), alcohol consumption (frequency, quantity, and type of drinks [36]) and smoking (frequency and quantity), urbanity [37], utilization of the health system (standardized single questions on distance to and consultations of general practitioner, medical specialists and hospital, utilization of health care programs, health insurance [38, 39]), and changes in health behavior after part A, as well as validated questionnaires for comorbidity (CCI) [44], depression (PHQ-8) [45], amnestic mild cognitive impairment (TICS-M) [46], social support (BSSS) [47], stress perception (PSS) [40], childhood trauma (CTS) [48], affect (PANAS) [49], psychological well-being (PWB) [50], musculoskeletal complaints such as (back) pain (CPG, BPI) [51–53], and the risk of developing chronic pain (RSI) [35]. Trained interviewers make an appointment in advance to guarantee a quiet setting for the telephone interview that is limited to 30 min.

### Consulting on nutrition and physical activity

After finalization of part A routine, the participants are advised verbally and by a flyer on recommendations about healthy nutrition and regular physical activity to prevent either the MetS (primary prevention) or the development or manifestation of its secondary diseases (secondary and tertiary prevention). Information is provided by the health care professional and is based on the recommendations of the German Nutrition Society (e.g., eat several servings of vegetables and fruits daily; prefer whole grain products; reduce sugar and salt intake; prefer vegetable oils; drink water or other calorie-free beverages) [57], as well as the WHO guideline for health-promoting physical activity (e.g., do at least 150 min of moderate-intensity aerobic physical activity per week; muscle-strengthening activities involving major muscle groups on 2 or more days per week) [58].

In part B, the participants receive individualized nutritional counseling from the physician. In addition, some simple sports exercises are demonstrated by the trained health care professional directly on site. These exercises use the participant’s own body weight, address core, and extremity strength (e.g., bridging, heel drop exercise, air cycling, wall push-ups) and can be performed independently after brief instruction. The practical instructions are supported by an illustrated flyer.

### Outcomes

Outcome of the pilot study is to evaluate the transfer of clinical, stationary routines into mobile examination units and to test the reliability and validity of the diagnosis of MetS and its secondary diseases in the mobile setting

In study part A, the “Mobile Brandenburg Cohort” is established by gathering a demographically representative study population via spontaneous recruitment. Central outcome of study part A is the prevalence of MetS and its components in this (rural) sample in Brandenburg. Main outcome of study part B is the validation of the MetS screening by medical diagnosis as well as the prevalence of its previously undiagnosed secondary diseases.

To reveal causal, risk, and moderating factors of MetS in Brandenburg, possible associations of MetS with socio-demographic data, psychometric parameters, and life style factors (e.g., socio-economic status, urbanity, social support, physical activity, perceived stress, life events; part A) as well as clinical and psychological parameters (e.g., comorbidities; part B) will be investigated.

The feasibility of the mobile measurements for MetS (part A) and secondary diseases (part B) in the patient-centered (mobile) setting will be evaluated. The following criteria are defined for success: In the pilot study, the mobile setup is evaluated by valid and reliable measurements of the MetS components with point-of-care analyzers applying conventional scientific criteria. The established diagnostics procedure is tailored to take maximal 20 min (on average) for part A and 75 min (on average) for part B and will be examined. For part A, at least 15 participants per measurement day, and for part B, at least 4 participants per measurement day are defined as a success. The feasibility assessment encompasses also qualitative criteria to evaluate an optimal balance between effort (approach, staff) and benefit (participation in the study).

By determining the rates of newly identified cases of MetS (part A) and indications for secondary diseases (part B), we will determine the usefulness of the mobile laboratory developed in the pilot study. This will help to reveal new ways for early diagnostic and prevention of secondary diseases in a patient-centered (mobile) care setting.

### Data management and statistics

The study is conducted in compliance with the EU’s General Data Protection Regulation (GDPR) [59]. Participant’s data will be processed pseudonymized. All data will be first documented in paper-pencil form and then entered in an electronic data base for further analyses. Programed plausibility checks (including, i.e., valid value checks and range checks) will be performed after assessment completion. All data are to be evaluated descriptively (i.e., mean, standard deviation, median, ranges, frequencies) to evaluate prevalence of MetS and of the respective risk factors (in the full study cohort and in defined subgroups, e.g., according to age and recruitment regions). The data sets will be evaluated conducting full case and complete case analyses. Paired *t* tests and Pearson’s Chi-squared tests are planned to evaluate any gender differences to all predictor and outcome variables. To evaluate the association of MetS with other assessed factors and variables (e.g., anthropometric parameters, age, sex, urbanity, physical activity, stress perception, social support, identified abnormalities, or diseases), inferential statistics (e.g., regression analysis, ANOVA), and cluster analyses are planned.

## Discussion

The study presented in this protocol will test the feasibility of new approach for mobile and near-to-home diagnostic to realize an early detection of risk factors and diseases linked to MetS in order to implement prevention strategies to reduce the morbidity and mortality of the population, especially in rural areas. The mobile setup will include time-, space-, and cost-efficient procedures which are to be predominantly performed by health care professionals (and physician-independent) and will lead to valid and reliable diagnostics of MetS and its pathophysiological consequences, which will be qualitatively equivalent to routine stationary examinations. In contrast to other mobile care and prevention ventures (e.g., mobile mammography van), walk-ins and spontaneous consultations enables a comprehensive access to precautionary and preventive health care.

According to the European policy for health and well-being [60], this mobile diagnostic approach should contribute to reduce health inequalities, strengthen public health, and ensure people-centered health systems and low-threshold access to prevention and health care. It should provide a complementary strategy for visiting a general practitioner and local health care providers. Thus, the mobile examination is the preliminary stage that enables an early connection to the health care system, if necessary. Individuals can be identified as persons at risk even before the onset of any disease and thus early prevention strategies can be initiated. Hence, only individuals who have been identified as requiring further diagnosis and treatment are referred to the relevant regional providers and consequently, only patients at risk and those with diseases are treated earlier, while persons without a risk profile are advised directly on primary prevention. In the long term, this strategy could complement the approaches discussed so far to increase medical capacity, reduce the physician shortage, and relieve the burden of the public health system [20, 61, 62].

The study presented here is carried out to target the MetS as one of the most important and prevalent risk factor complexes for numerous secondary diseases. Brandenburg, a rural and structurally weak region, possesses one of the highest prevalences of MetS [19] and one of the lowest patient-to-physician ratio nationwide [63]. By recruiting people from different regions, especially rural areas, and due to assessment of moderating factors, a unique data base and cohort will be established that will deliver important findings on prevalence and causes as well as influencing factors of MetS in Brandenburg. However, the concept is also transferable to other risk factor profiles and diseases as well as other (rural) regions, nationally and internationally. To this end, the established mobile diagnostics setup can be further developed and expanded by mobilizing already validated methods as well as by technical miniaturization. In addition, mobile prevention could be achieved by combining the mobile diagnostic approach with home-based intervention programs (especially, nutrition and exercise programs that can be easily integrated into everyday life) including monitoring of lifestyle factors by means of wearables and mobile applications. Moreover, these methods could be combined with a system for health data management and health data processing, enabling the prediction of risk factor profiles or to indicate appropriate prevention strategies depending on the mobile collected health data to formulate individual recommendations. Together with a network of local health care providers including clinics, health insurance companies, general practitioners, and medical specialists as well as due to cooperation with sports associations, sports clubs, and health facilities, an effective concept of early mobile diagnostic, prevention, treatment, and rehabilitation could built up. In the long-term, the mobile diagnostic approach and thereby the implementation of primary, secondary, and tertiary prevention strategies may help to reduce the disease burden and consequently the disease-specific mortality of the population, especially in rural and structurally weak areas.

### Study status

Participants must be recruited and enrolled for each of the three study parts. Recruitment for the pilot study has been completed with 50 participants. Planned recruitment and measurement days for main study part A in 2020 had to be canceled due to the COVID-19 pandemic. Therefore, recruitment for study part A has ended for the time being with a total of 3.931 participants enrolled. Recruitment for study part B is ongoing and not yet completed. For the mobile examination, 93 participants have been enrolled by beginning of 2020. Due to the COVID-19 pandemic, the focus is on recruitment for the telephone interviews of study part B, for which 94 participants have been enrolled so far.

## Data Availability

Not applicable.

## Abbreviations

ECG: Electrocardiogram
HbA1c: Glycated hemoglobin
HDL: High density lipoprotein
MetS: Metabolic syndrome
